# Protective Immunity against *Vibrio harveyi* in Grouper Induced by Single Vaccination with Poly (Lactide-co-glycolide) Microparticles Releasing Pleurocidin Peptide and Recombinant Glyceraldehyde-3-phosphate Dehydrogenase

**DOI:** 10.3390/vaccines8010033

**Published:** 2020-01-19

**Authors:** Shang-Pin Liu, Shu-Chun Chuang, Chung-Da Yang

**Affiliations:** 1 Department of Biological Science and Technology, College of Health and Nursing, Meiho University, 23 Pingguang Road, Neipu, Pingtung 912, Taiwan; x00008260@meiho.edu.tw; 2 Orthopaedic Research Center, Kaohsiung Medical University, No. 100, Shih-Chuan 1st Road, Kaohsiung 807, Taiwan; f86225016@ntu.edu.tw; 3 Regenerative Medicine and Cell Therapy Research Center, Kaohsiung Medical University, No. 100, Shih-Chuan 1st Road, Kaohsiung 807, Taiwan; 4 Graduate Institute of Animal Vaccine Technology, College of Veterinary Medicine, National Pingtung University of Science and Technology, No. 1, Shuefu Road, Neipu, Pingtung 912, Taiwan

**Keywords:** *Vibrio harveyi* (*V. harveyi*), single-dose vaccine, pleurocidin (PLE), recombinant glyceraldehyde-3-phosphate dehydrogenase (rGAPDH), poly(lactide-co-glycolide) (PLG), PLG-encapsulated PLE plus rGAPDH microparticles (PLG-PLE/rGAPDH MPs)

## Abstract

The peptide adjuvant, pleurocidin (PLE), and the *Vibrio harvey*i antigen, recombinant glyceraldehyde-3-phosphate dehydrogenase (rGAPDH) protein, were encapsulated with poly (lactide-co-glycolide) (PLG) polymers in our previous study to produce PLG-encapsulated PLE plus rGAPDH microparticles (PLG-PLE/rGAPDH MPs) that sustained stable release of both PLE and rGAPDH as well as, after two-time vaccination with MPs, generated long-term protective immunity against *V. harveyi* in grouper. Stable controlled-release of PLE plus rGAPDH from PLG-PLE/rGAPDH MPs is an attractive feature for developing an effective single-dose vaccine. In the present study, therefore, we aim to evaluate whether single administration with PLG-PLE/rGAPDH MPs in grouper would result in protective immunity against *V. harveyi*. Peritoneal vaccination of grouper with one dose of PLG-PLE/rGAPDH MPs raised serum titers over a long 12-week period. Moreover, twelve weeks after vaccination, significant lymphocyte proliferation and maximum TNF-α production were found in grouper immunized with a single dose of PLG-PLE/rGAPDH MPs. More importantly, immune responses elicited by single vaccination with PLG-PLE/rGAPDH MPs protected 80% of fish against a lethal peritoneal challenge of the highly virulent *V. harveyi* (Vh MML-1). In conclusion, our data truly reveal the feasibility of the development of a single-dose vaccine against *V. harveyi* based on PLG-PLE/rGAPDH MPs.

## 1. Introduction

*Vibrio harveyi* is a Gram-negative bacterium and has been reported to be a serious pathogenic bacterium for many marine vertebrates, including cultured fish species such as gilthead sea bream, sea bass, grouper, and salmonid fish [1], as well as invertebrates, such as lobster and shrimp [1,2]. Eye lesions, deep skin lesions, and ulcers as well as gastroenteric disorders are the obvious pathological changes in infected fish [1,3]. *V. harveyi* is the major etiological agent of vibriosis in grouper, an aquacultured fish species with high economic value in Southeast Asia, including Taiwan and China, to result in high morbidity and mortality in grouper and further induce a significant economic loss in the grouper aquaculture [4]. Conventional antibiotics have been used in grouper farms to control vibriosis. However, the overuse of these antibiotics gives rise to the emergence of resistant bacteria as well as the issue of the drug residue in fish and the environment [5]. Vaccination in fish has been considered to be a possible option to alleviate such threats induced by pathogenic bacteria and antibiotic use [6,7].

Since antigenic epitopes displayed by the outer membrane proteins (OMPs) on the surface of pathogenic bacteria can be favorably recognized as foreign substances by the host immune system [8,9], the development of fish vaccines against *V. harveyi* has been recently focused mainly on these OMPs [8,9,10,11,12,13]. In bacteria, the glyceraldehyde-3-phosphate dehydrogenase (GAPDH), a crucial enzyme in glycolysis, can be expressed on the outer membrane to play an important role in the bacterial infection process [14]. Moreover, GAPDH has been demonstrated to display its immunogenicity to result in anti-*V. harveyi* protective immunity in fish [8,9,10]. Therefore, GAPDH seems more likely to be a proper antigen candidate to explore the development of effective vaccines against *V. harveyi*. However, potent adjuvants that can strengthen immunity and protection against aquatic infectious diseases have recently drawn great attention in developing fish vaccines [15,16,17]. If efficacious adjuvants are applied in the GAPDH-based vaccine formulation, the immunogenicity of GAPDH can be enhanced to further improve anti-*V. harveyi* protective immunity in fish. Since cationic antimicrobial peptides (AMPs), especially pleurocidin (PLE) peptide [18,19,20,21], have shown their capabilities to activate both innate and adaptive immune responses [22,23], there has been a great interest in developing these peptides as strong adjuvants in numerous studies [15,16]. Therefore, the PLE peptide-induced immunomodulatory effects make PLE peptide a particular substance to be explored as a peptide adjuvant. On the other hand, biodegradable microparticles (MPs) prepared from poly(lactide-co-glycolide) (PLG) polymers have been conducted as powerful delivery systems to encapsulate antigens for generating the PLG MP vaccines that can sustain release of antigens for a long period [16,17]. Such controlled-release of antigens is a particularly attractive characteristic for developing single-dose vaccines without additional administration of booster doses [24,25,26].

In our previous study, we had encapsulated both the peptide adjuvant, pleurocidin, and the *V. harvey*i antigen, *E. coli (Escherichia coli*)-based recombinant GAPDH (rGAPDH) protein, into PLG MPs to prepare PLG-encapsulated PLE plus rGAPDH (PLG-PLE/rGAPDH) MPs [8]. We found that PLG-PLE/rGAPDH microparticles could perform 30-day sustained release of PLE and rGAPDH to raise grouper protective immunity against an experimental challenge of low virulent *V. harveyi* (BCRC13812) following two peritoneal shots of microparticles (85%) [8]. However, vaccination with multi-dose vaccines to accomplish protective immunity is usually cost-ineffective, complex and its compliance is often difficult for use in complete vaccination of food animals [24,25,26]. Actually, the sustained release of PLE and rGAPDH we found in our previous study [8] is a promising characteristic for developing a single-dose vaccine. Therefore, it would be certainly worthy of investigation to elucidate the feasibility of the development of a single-dose vaccine against *V. harveyi* in grouper based on PLG-PLE/rGAPDH MPs. In the present study, therefore, we aimed to evaluate whether immunity induced by single peritoneal vaccination with PLG-PLE/rGAPDH MPs could also protect grouper from a lethal challenge of high virulent *V. harveyi* (Vh MML-1).

## 2. Materials and Methods

### 2.1. Bacterial Culture

The Vh MML-1 strain of *V. harveyi* was isolated from diseased grouper collected from a fish farm (22°26′ N, 120°30′ E) in southern Taiwan. The virulence of Vh MML-1 strain had been determined to be a highly virulent strain because 100% mortality rate in grouper by 11 days could be achieved by peritoneal infection with 1.2 × 10^6^ CFU (colony-forming unit) of *V. harveyi* (Vh MML-1), which is an amount two times higher than LD_50_ (6 × 10^5^ CFU) [8,27]. *V. harveyi* was cultured in tryptic soy broth (TSB; BD, Franklin Lakes, NJ, USA) with 2% NaCl at 25 °C for 18 h to mid-logarithmic phase [8,27].

### 2.2. Preparation of PLG-PLE/rGAPDH MPs

The PLG-PLE/rGAPDH MPs utilized for vaccinating grouper in the present study were produced through the water/oil/water double emulsion method and their characteristics were analyzed according to the procedures in our previous study [8]. Briefly, 20 mL of a 10% solution of 50:50 PLG (Sigma, St. Louis, MO, USA) in dichloromethane (Sigma, St. Louis, MO, USA) was mixed with 2 mL of a solution containing PLE (0.5 mg/mL) plus rGAPDH (5 mg/mL) by using a PRO200 homogenizer (PRO Scientific, Oxford, OH, USA) equipped with 10 mm × 150 mm generator at 10,000 rpm for 5 min to produce a water/oil emulsion. The resulting emulsion was further homogenized with 20 mL of a 2% polyvinyl alcohol (Sigma, St. Louis, MO, USA) solution at 10,000 rpm for 5 min to generate a stable water/oil/water emulsion. The water/oil/water emulsion was then stirred for 18 h at room temperature (RT) and pressurized to promote solvent evaporation and the formation of PLG-PLE/rGAPDH MPs in a laboratory fume hood. These microparticles were collected by centrifugation at 4000× *g* for 30 min, washed three times with distilled water to remove non-entrapped protein/peptide, and then lyophilized by an FD-5030 freeze dryer (Panchum, Kaohsiung, Taiwan) for storage at −20 °C. The particle size was determined by an N5 submicron particle size analyzer (Beckman Coulter, Brea, CA, USA). The resulting PLG-PLE/rGAPDH MPs, 3.21~6.27 μm in diameter, displayed 72~83% entrapment efficiency and their cumulative release of PLE plus rGAPDH in PBS gradually increased over a 30-day period [8].

### 2.3. Inactivated V. harveyi Bacteria

The inactivated *V. harveyi* bacteria were prepared according to our previous studies [8,27]. The highly virulent Vh MML-1 strain of *V. harveyi* was grown overnight on tryptic soy agar (TSA) with 2% NaCl at 25 °C. *V. harveyi* single colony was then picked up from the agar plate and cultured in 5 mL of TSB with 2% NaCl at 25 °C for 18 h. Afterwards, further expand culture to an OD_600_ of 1 was undertaken in 500 mL of TSB with 2% NaCl. The cultured bacteria were then inactivated with 0.3% (v/v) of formalin for 24 h and washed three times with PBS to remove residual formalin. The bacterial pellet was re-suspended in 40 mL of PBS. In order to confirm the inactivation of bacteria, 0.1 mL of the resulting inactivated bacteria suspension was plated on TSA with 2% NaCl and no colony was present on TSA after growth at 25 °C overnight. The resulting suspension of inactivated *V. harveyi* bacteria was then stored at 4 °C until use.

### 2.4. Bacterial Lysate

The bacterial lysate was produced from the highly virulent Vh MML-1 strain of *V. harveyi* as described previously [8,27]. *V. harveyi* (Vh MML-1) was grown in 50 mL of TSB with 2% NaCl at 25 °C to an OD_600_ of 1. The cultured *V. harveyi* bacteria were spun down by centrifugation at 3000× *g* for 10 min and then washed three times with saline. Afterwards, bacteria were re-suspended in 2 mL saline, sonicated by an ultrasonic processor VCX 130 (Sonics, Newtown, CT, USA) and then centrifuged at 12,000× *g* for 30 min at 4 °C. The resulting soluble supernatant was collected to remove its residual lipopolysaccharide (LPS) by the Detoxi-Gel Endotoxin Removing column (Thermo Scientific, Waltham, MA, USA) and the level of LPS (below 0.1 EU/mL) was confirmed by the Pierce LAL Chromogenic Endotoxin Quantitation Kit (Thermo Scientific, Waltham, MA, USA) [28]. The protein concentration of the bacterial lysate (soluble supernatant) was measured using the dye-binding DC protein assay (Bio-Rad, Hercules, CA, USA) with bovine serum albumin (BSA) as a standard. Aliquots of the bacterial lysate were stored at −20 °C until use.

### 2.5. Animals

Grouper (*Epinephelus coioides*), weighing 52 ± 6 g, were ordered from a disease-free grouper farm in southern Taiwan. Fish were maintained in high containment facilities and were reared at 25 °C in 300 L fiberglass-reinforced plastics (FRP) tanks with filtered and aerated re-circulating seawater. Fish were fed with commercial dry pellets (crude protein (above 50%), crude fat (above 8.4%), crude fiber (under 2.9%), ash (under 16.0%) and moisture (under 10%)) (Hai-Yu, Kaohsiung, Taiwan) twice a day. The fish health status was checked every day. After one week, fish were ready for experimental use. The Institutional Animal Care and Use Committee (IACUC), National Pingtung University of Science and Technology reviewed and approved all processes of fish administrations (NPUST-105-023).

### 2.6. Immunization

Six groups of 25 grouper each were peritoneally vaccinated once with 10 μg of PLG-PLE/rGAPDH MPs, 1 × 10^8^ CFU of inactivated *V. harveyi* bacteria formulated with the Montanide ISA 763 AVG adjuvant (Seppic, Paris, France) (Inactivated bacteria (ISA 763 AVG)), 10 μg of rGAPDH, 10 μg of PLE, 10 μg of PLG empty MPs or PBS. Specific immune responses against *V. harveyi* were examined by the following immunoassays.

### 2.7. Serum Assay

Three weeks after peritoneal vaccination, the antigenic specificity of sera collected from vaccinated grouper was assessed by Western blot [8]. Moreover, grouper sera were collected at weeks 0, 3, 6, 9, and 12 and their anti-*V. harveyi* titers were determined by ELISA as described previously [8]. Each well of 96-well flat-bottomed polystyrene microplates (Nunc, Rochester, NY, USA) was coated with 10 μg/mL of *V. harveyi* lysate in 100 μL of 0.1 M carbonate/bicarbonate buffer (pH 9.4) and incubated overnight at 4 °C. The wells were then washed with PBS and blocked with blocking buffer (PBS containing 5% BSA). Samples of 50-time diluted serum in serial dilution were added to the wells (50 μL/well) and incubated for 1.5 h at 37 °C. After washing three times with PBST (PBS with 0.05% Tween 20), the wells were incubated with 1:1000-diluted guinea pig anti-grouper immunoglobulin sera for 1 h at 37 °C. PBST washes were carried out again, and each well was incubated with 50 μL of biotinylated goat anti-guinea pig IgG (Vector Laboratories, Burlingame, CA, USA) diluted in the blocking buffer (1:3000) for 1 h at 37 °C. After washing with PBST, 50 μL of streptavidin/peroxidase (1:3000 dilution) was added to each well. After incubation for 1 h at room temperature, color development and serum titer determination were then performed as described previously [8].

### 2.8. Lymphocyte Proliferation Assay

Twelve weeks after peritoneal vaccination, three grouper from each group were euthanized to collect head kidney lymphocytes as described previously [8,27]. Then, 2 × 10^5^ lymphocytes suspended in 200 μL of L-15 culture medium (CM) were seeded in each well of 96-well culture plates, stimulated with *V. harveyi* lysate (20 μg/mL) and then incubated for 72 h at 25 °C as described previously [8,27]. In addition, Con A (10 μg/mL)- and CM-treated cultures were respectively conducted to use as positive and negative controls. Then, the BrdU (5-bromo-2′-deoxyuridine) Colorimetric Cell Proliferation ELISA Kit (Roche, Basel, Switzerland) was utilized to measure grouper lymphocyte proliferation according to the manufacturer’s instructions. Finally, the stimulation index ($\mathrm{SI}\;=\;\frac{{\mathrm{OD}}_{450}\;\mathrm{values}\;\mathrm{from}\;\mathrm{lysate}\text{-}\mathrm{treated}\;\mathrm{culture}\;\mathrm{or}\;\mathrm{Con}\;A\text{-}\mathrm{treated}\;\mathrm{cultures}}{{\mathrm{OD}}_{450}\;\mathrm{values}\;\mathrm{from}\;\mathrm{CM}\text{-}\mathrm{treated}\;\mathrm{cultures}}$) for each group was counted as described previously [8,27].

### 2.9. Quantitative Real-Time Reverse Transcriptase-PCR (qRT-PCR)

Twelve weeks after peritoneal vaccination, the expression levels of TNF-α gene in grouper lymphocytes under the stimulation of bacterial lysate were analyzed by real-time PCR. Briefly, grouper lymphocytes were collected from different groups and 1 × 10^6^ lymphocytes in 2 mL of L-15 culture medium (CM) were seeded in each well of 6-well culture plates. After overnight culture, lymphocytes were treated with *V. harveyi* lysate (20 μg/mL) or Con A (10 μg/mL) (Sigma, St. Louis, MO, USA) for another 6 h. The Con A- and CM-treated cultures were respectively conducted as positive and negative controls. After incubation, total cellular RNA was extracted by TRIzol™ reagent (Invitrogen, Waltham, MA, USA) from the treated lymphocytes to reversely transcribe (500 ng of RNA) into cDNA by SuperScript III™ reverse transcriptase (Invitrogen, Waltham, MA, USA) as described previously [26]. The induced expression levels of grouper TNF-α gene were then analyzed by real-time PCR in a LightCycler instrument with use of a SYBR Green system (Roche, Basel, Switzerland). Primers for TNF-α gene (TNF-α (F)/(R)) [29] and β actin gene (β actin (F)/(R)) [30] are shown in Table 1. The β actin gene expression was conducted as the internal reference. A 20-μL reaction mixture consisted of the FastStart DNA Master SYBR Green I Kit (Roche, Basel, Switzerland), 0.5 μM of primers and 2 μL of 5-time diluted cDNA prepared as described above was subjected to an initial denaturation at 94 °C for 2 min followed by 40 cycles of 94 °C for 15 s (denaturation), 57 °C for 15 s (annealing), and 72 °C for 20 s (extension). After each extension, the amount of PCR product was assessed by detecting the fluorescence of SYBR Green I attached to amplification product. LightCycler software, version 3.0 (Roche, Basel, Switzerland) was then utilized to analyze fluorescence curves. Finally, results of TNF-α gene expression was presented as the fold change ($\frac{\mathrm{The}\;\mathrm{quantity}\;\mathrm{of}\;\mathrm{specific}\;\mathrm{TNF}\text{-}\alpha \;\mathrm{mRNA}\;\mathrm{from}\;\mathrm{stimulated}\;\mathrm{cells}}{\mathrm{The}\;\mathrm{quantity}\;\mathrm{of}\;\mathrm{TNF}\text{-}\alpha \;\mathrm{mRNA}\;\mathrm{from}\;\mathrm{unstimulated}\;\mathrm{cells}}$) as described previously [26]. All samples were processed in triplicate.

### 2.10. Bacterial Challenge

Twelve weeks after peritoneal vaccination, six groups of 20 fish each were challenged with a peritoneal injection of 6 × 10^6^ CFU of the highly virulent *V. harveyi* (Vh MML-1) in order to assess whether the induced immune responses could protect fish against *V. harveyi* challenge. The challenge dose was 10 times higher than LD_50_ (6 × 10^5^ CFU) that we had previously determined according to the previous process [8,27]. Fish were observed every day for another 28 days and deaths were recorded as they occurred. Final survival rates of different groups were counted as described previously [8,27].

### 2.11. Statistical Analysis

According to our previous studies [8,26,27,28,31,32], the results obtained in the present study were statistically analyzed as follows. The nested design was used to statistically compare the serum titers (log_10_) from different groups and least significant difference (LSD) multiple comparison was used to test the means at different time points in each group. One-way ANOVA was used to statistically compare SI values and TNF-α fold changes. The Chi-square test was used to analyze the survival rates of different groups. A *p*-value of less than 0.05 was considered to be significant.

## 3. Results

### 3.1. Antigenic Specificity of Grouper Antisera Following Single Immunization

**Figure 1:**
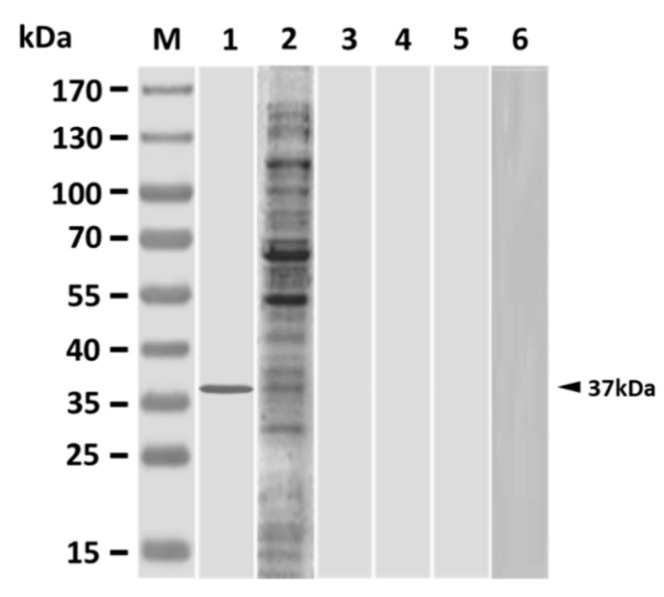
Antigenic specificity of sera from vaccinated grouper. Three weeks after vaccination, *V. harveyi* lysate was analyzed with sera from grouper peritoneally vaccinated with a single shot of PLG-encapsulated PLE plus rGAPDH microparticles (PLG-PLE/rGAPDH MPs) (lane 1), a single shot of inactivated bacteria (ISA 763 AVG) (lane 2), a single shot of rGAPDH (lane 3), a single shot of PLE (lane 4), a single shot of PLG empty MPs (lane 5), or a single shot of PBS (lane 6). Standard protein markers (lane M) are shown at the left.

Results showed that single peritoneal vaccination with PLG-PLE/rGAPDH MPs could produce serum antibodies to recognize the native GAPDH (37kDa) in bacterial lysate (Figure 1, lane 1). On the other hand, one shot of the oil formulation, inactivated bacteria (ISA 763 AVG), also induced antibodies to recognize numerous proteins in lysate (Figure 1, lane 2). However, peritoneal immunization with one shot of rGAPDH, PLE, PLG empty MPs or PBS did not generate serum antibodies to react to any proteins in lysate (Figure 1, lanes 3~6). Thus, both PLG-PLE/rGAPDH MPs and inactivated bacteria (ISA 763 AVG) could elicit antibodies in the grouper following single peritoneal vaccination.

### 3.2. Sustained High Serum Titers Raised by Single Vaccination with PLG-PLE/rGAPDH MPs

**Figure 2:**
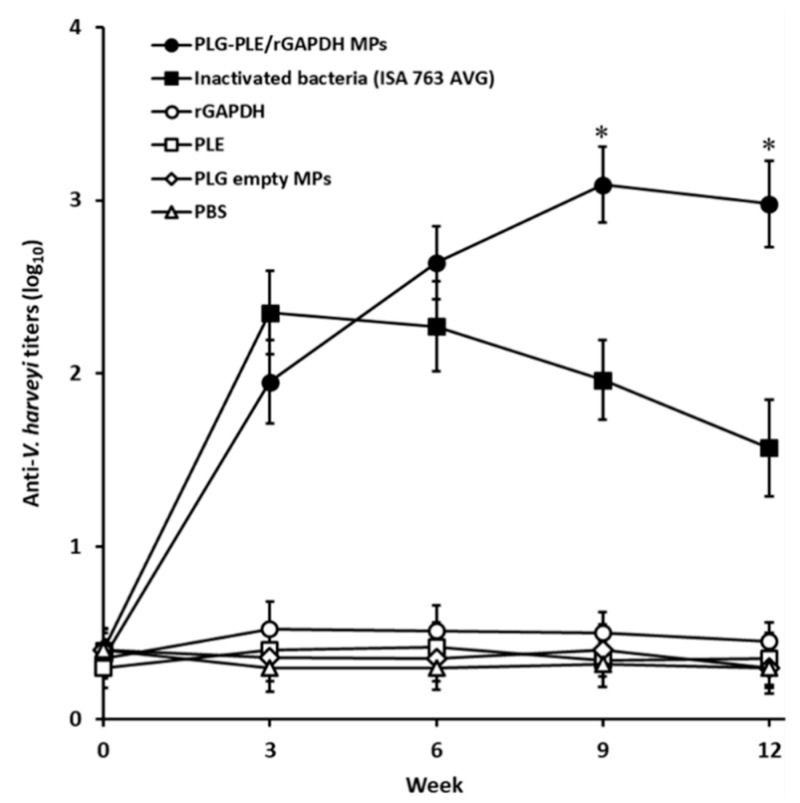
Anti-*V. harveyi* serum titers of vaccinated grouper. Six groups of grouper were peritoneally vaccinated with a single shot of PLG-PLE/rGAPDH MPs (●), a single shot of inactivated bacteria (ISA 763 AVG) (■), a single shot of rGAPDH (○), a single shot of PLE (□), a single shot of PLG empty MPs (◊) or a single shot of PBS (∆). Following peritoneal vaccination, anti-*V. harveyi* serum titers of different groups were examined by ELISA every three weeks. Results were expressed as the mean of log_10_ titers ± standard deviation (SD). * A significant difference (*p* < 0.05) exists when comparing the PLG-PLE/rGAPDH MPs group to the inactivated bacteria (ISA 763 AVG) group.

Antiserum titers of vaccinated grouper are shown in Figure 2. Similarly low antiserum titers were found in different fish groups before immunization (week 0). After single peritoneal vaccination with PLG-PLE/rGAPDH MPs, antiserum titers raised from week 0 to week 9 (*p <* 0.05, LSD multiple comparison) and then insignificantly reduced from week 9 to week 12 (*p >* 0.05, LSD multiple comparison). Thus, peritoneal vaccination with a single shot of PLG-PLE/rGAPDH MPs led to long-lasting anti-*V. harveyi* serum titers in grouper. Although high serum titers in grouper were also elicited by one shot of inactivated bacteria (ISA 763 AVG) in the first three weeks (*p <* 0.05, LSD multiple comparison), they gradually reduced from week 3 to week 12 (*p <* 0.05, LSD multiple comparison). In further comparison, following single peritoneal vaccination in grouper, PLG-PLE/rGAPDH MPs and inactivated bacteria (ISA 763 AVG) resulted in similar serum titers in the first six weeks (*p >* 0.05, Nested design). More importantly, starting from the 6th week to the 12th week, grouper serum titers produced by single peritoneal vaccination with PLG-PLE/rGAPDH MPs remained significantly higher than those raised by one peritoneal shot of inactivated bacteria (ISA 763 AVG) (*p <* 0.05, Nested design). In contrast, single peritoneal vaccination of grouper with rGAPDH, PLE, PLG empty MPs or PBS led to very little anti-*V. harveyi* serum titers during a 12-week period. Therefore, sustained high anti-*V. harveyi* serum titers in grouper could be generated by peritoneal administration with a single dose of PLG-PLE/rGAPDH MPs.

### 3.3. Robust Lymphocyte Proliferation Produced by Single Vaccination with PLG-PLE/rGAPDH MPs

**Figure 3:**
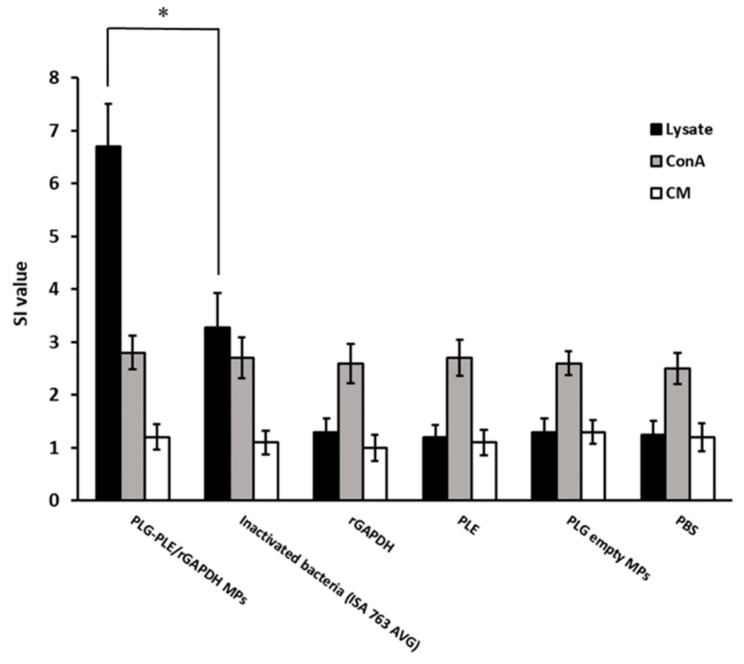
Grouper lymphocyte proliferation to *V. harveyi* lysate. Six groups of grouper were peritoneally vaccinated with a single shot of PLG-PLE/rGAPDH MPs, a single shot of inactivated bacteria (ISA 763 AVG), a single shot of rGAPDH, a single shot of PLE, a single shot of PLG empty MPs, or a single shot of PBS. Twelve weeks after peritoneal vaccination, proliferative responses of lymphocytes stimulated with *V. harveyi* lysate (■), Con A (■), or culture medium (CM) (□) were assessed and expressed as stimulation index (SI) values. Results were presented as the mean of SI values ± standard deviation (SD). * A significant difference (*p* < 0.05) exists when comparing the PLG-PLE/rGAPDH MPs group to the inactivated bacteria (ISA 763 AVG) group.

Grouper lymphocyte proliferation to *V. harveyi* lysate was shown in Figure 3. Under the stimulation of *V. harveyi* lysate, one dose of PLG-PLE/rGAPDH MPs led to significantly higher SI values than one dose of inactivated bacteria (ISA 763 AVG) (*p <* 0.05, one-way ANOVA). In contrast, one shot of rGAPDH, PLE, PLG empty MPs or PBS caused little, if any, proliferation to *V. harveyi* lysate in grouper. On the other hand, lymphocytes, from different fish groups, respectively stimulated with 10 μg/mL of Con A or culture medium (CM) were found to proliferate to a similar extent (*p >* 0.05, one-way ANOVA). Thus, twelve weeks following single peritoneal immunization, PLG-PLE/rGAPDH MPs releasing PLE plus rGAPDH still could enhance anti-*V. harveyi* lymphocyte proliferation in grouper.

### 3.4. High TNF-α Production Following Single Vaccination with PLG-PLE/rGAPDH MPs 

In further comparison, TNF-α production caused by single vaccination with PLG-PLE/rGAPDH MPs was significantly higher than that raised by single vaccination with inactivated bacteria (ISA 763 AVG) (*p <* 0.05, one-way ANOVA) (Table 2). Similar low fold changes to bacterial lysate were detected in lymphocytes collected from grouper given one shot of rGAPDH, PLE, PLG empty MPs, or PBS (*p >* 0.05, one-way ANOVA) (Table 2). Under Con A stimulation, grouper lymphocytes, from different fish groups, generated similar TNF-α fold changes (*p >* 0.05, one-way ANOVA). Therefore, significant TNF-α production in grouper could be smoothly induced by peritoneal vaccination with one shot of PLG-PLE/rGAPDH MPs.

### 3.5. Strong Protection against V. harveyi in Grouper Induced by Single Vaccination with PLG-PLE/rGAPDH MPs

**Figure 4:**
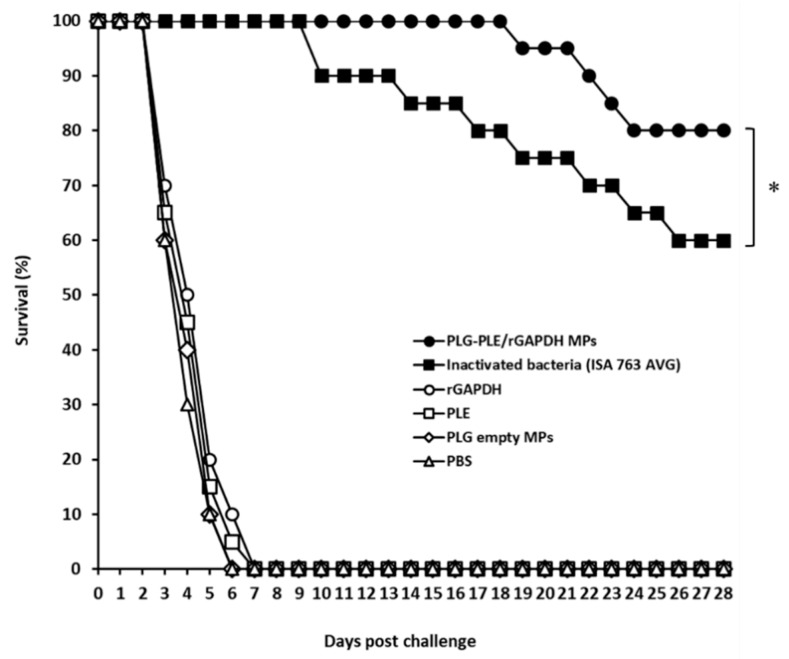
Grouper survival after a lethal peritoneal challenge of the highly virulent *V. harveyi* (Vh MML-1). Grouper were peritoneally vaccinated with a single shot of PLG-PLE/rGAPDH MPs (●), a single shot of inactivated bacteria (ISA 763 AVG) (■), a single shot of rGAPDH (○), a single shot of PLE (□), a single shot of PLG empty MPs (◊), or a single shot of PBS (∆). Twelve weeks after peritoneal vaccination, six groups of 20 grouper each were infected with 6 × 10^6^ CFU of *V. harveyi* (Vh MML-1) via the peritoneal route. Fish were observed for another 28 days and final survival rates of different groups were counted. (*) indicates *p <* 0.05 when comparing the PLG-PLE/rGAPDH MPs group to the inactivated bacteria (ISA 763 AVG) group.

After challenge, the survival rate in each group was recorded (Figure 4). Sixteen out of twenty grouper given one peritoneal shot of PLG-PLE/rGAPDH MPs survived during the challenge study and showed a high protection of 80%. A moderate protection of 60% following challenge was found in fish received a single peritoneal injection of inactivated bacteria (ISA 763 AVG). In comparison with the inactivated bacteria (ISA 763 AVG) group, single peritoneal immunization with PLG-PLE/rGAPDH MPs in grouper increased the survival rate by 20%. Therefore, following single peritoneal vaccination, PLG-PLE/rGAPDH MPs resulted in significantly higher protection in grouper than in inactivated bacteria (ISA 763 AVG) (*p <* 0.05, Chi-square test). However, a single peritoneal shot of rGAPDH, PLE, PLG empty MPs or PBS did not generate protection against *V. harveyi* in grouper. These results indicated that immune responses produced by one shot of PLG-PLE/rGAPDH MPs through the peritoneal administration rendered substantial protection against the experimental *V. harveyi* challenge in grouper.

## 4. Discussion

Vaccination against aquatic bacterial diseases in aquaculture has allowed us to possibly prevent the disease outbreaks [6,7]. Although recent subunit vaccines against *V. harveyi* based on DNA engineering technology have been studied, they have produced different protective abilities against experimental lethal infections of the virulent *V. harveyi* strains [9,10,11,12,13]. Generally, vaccines comprising purified recombinant proteins like rGAPDH are weakly immunogenic and therefore require potent adjuvants to assist them to give rise to strong immunity and protection [15,16,17]. In our previous study, the PLE peptide has been demonstrated to exercise an adjuvant effect on improving immunogenicity of the rGAPDH protein encapsulated into PLG MPs [8]. Although, in the same study, two peritoneal shots of PLG-PLE/rGAPDH MPs capable of sustaining stable release of PLE and rGAPDH have elicited protective immunity against low virulent *V. harveyi* (BCRC13812) in grouper, we still need an anti-*V. harveyi* single-dose vaccine used in fish to solve the issues resulted from multi-dose vaccination. More importantly, the capability of PLG-PLE/rGAPDH MPs to regulate the stable release of both antigen and adjuvant would be favorable for its application in developing an efficacious single-dose vaccine against *V. harveyi*. Thus, in the present study, we continued to extend our previous finding about sustaining retention of PLE plus rGAPDH to assess the feasibility of the use of PLG-PLE/rGAPDH MPs as a single-dose vaccine against high virulent *V. harveyi* (Vh MML-1) in grouper.

In the present study, we found that peritoneal vaccination with a single shot of PLG-PLE/rGAPDH MPs in grouper caused long-lasting (12 weeks) high serum titers (Figure 2). Before challenge, both strong lymphocyte proliferation (Figure 3) and high TNF-α production (Table 2) were detected in grouper vaccinated with one shot of PLG-PLE/rGAPDH MPs. More importantly, 80% (16/20) of grouper given one shot of PLG-PLE/rGAPDH MPs survived at least 28 days after a lethal peritoneal challenge of the highly virulent *V. harveyi* (Figure 4). However, peritoneal vaccination with a single injection of inactivated bacteria emulsified with the Montanide ISA 763 AVG adjuvant was not able to maintain protective immunity against *V. harveyi* in grouper (Figure 2 and Figure 3). Peritoneal vaccination with one shot of the PLG MP vaccine therefore may be superior to one peritoneal injection of the oil formulation vaccine in eliciting long-lasting immunity against *V. harveyi*. Thus, an effective single-dose anti-*V. harveyi* vaccine based on the PLG encapsulation technique that is promised to undertake the sustained release of both rGAPDH and PLE from PLG MPs would be a crucial strategy for successfully controlling vibriosis in grouper. We have revealed the feasibility of utilizing PLG-PLE/rGAPDH MPs as a single-dose vaccine against *V. harveyi* in grouper in the present study.

The ability of MPs made from PLG to regulate the sustained release of entrapped vaccine antigens can maintain immune responses in animals following single immunization [24,25,26]. According to previous papers, PLG MPs may perform pulsed and/or slow release manner(s) of antigenic proteins to promote the immune system [33]. The PLG MP vaccines prepared according to our previous PLG encapsulation process have been demonstrated to carry out a triphasic controlled-release profile (in vitro in PBS) made up of an initial pulsed release, a very slow release, and a final pulsed release [28,31,32]. Actually, such a triphasic release model comes from the initial pulsed diffusion of protein absorbed onto the surface of PLG MPs, the very slow diffusion of encapsulated protein, and the final pulsed protein diffusion because of the degradation of PLG MPs [34,35,36]. Thus, our previous studies [28,31,32] and those recorded by others [34,35,36] imply us that the in vitro 30-day release of both PLE and rGAPDH from PLG-PLE/rGAPDH MPs seen in our previous study [8] might also perform a triphasic controlled-release profile, which possesses two pulsed and one slow release manners. However, more effort is further needed to confirm the release profile exercised by PLG-PLR/rGAPDH MPs. Since the 30-day release study in our previous study was done in PBS (in vitro), it may not entirely represent in vivo release in grouper. Anyway, the sustained high serum titers found in ELISA in the present study (Figure 2) revealed that the long-lasting release of PLE plus rGAPDH exercised by PLG-PLE/rGAPDH MPs effectively prolonged GAPDH-specific humoral immunity in grouper.

Based on previous investigations, PLG MP vaccines are capable of inducing both humoral and cell-mediated immunity (CMI) in animals [16,17]. Our recent study has shown that the lymphocyte proliferation response can be utilized to judge grouper CMI [8]. Moreover, TNF-α, a critical Th1-type cytokine, not only induces the inflammatory response in fish [37] but also performs an important biomarker for detecting fish health status and vaccine potency [38]. Therefore, we put efforts into analyzing these two activities, lymphocyte proliferation and TNF-α production, in the present study to evaluate whether protective Th1 CMI is induced. In the present study, twelve weeks following peritoneal vaccination, strong lymphocyte proliferation against *V. harveyi* lysate was readily detected in grouper given one shot of PLG-PLE/rGAPDH MPs (Figure 3). Moreover, before challenge, grouper vaccinated with a single peritoneal shot of PLG-PLE/rGAPDH MPs generated significant TNF-α production (Table 2). Therefore, a single shot of PLG-PLE/rGAPDH MPs enhanced Th1 CMI against *V. harveyi* in grouper. Indeed, the inflammatory response induced by TNF-α is like a two-edged sword and an appropriate response between pro-inflammatory and anti-inflammatory cytokines is necessary to prevent infections induced by pathogenic microbes [37]. Further studies are therefore required to analyze these cytokine profiles during vaccination. On the other hand, sustained high antiserum titers detected in the grouper after single peritoneal administration with PLG-PLE/rGAPDH MPs (Figure 2) certainly indicated that humoral immunity, which is modulated by Th2 cytokines, should contribute to protection against the *V. harveyi* challenge. Therefore, a single peritoneal injection of PLG-PLE/rGAPDH MPs resulted in mixed Th1/Th2 immunity against *V. harveyi* in grouper. 

Previous studies have indicated that antigenic variation depending on different strains and/or isolates of *V. harveyi* results in a difficulty in developing a cross-protective vaccine against *V. harveyi* [39]. Actually, the rGAPDH protein encapsulated in PLG MPs was prepared in our previous study by cloning the *gapdh* gene derived from the lowly virulent strain (BCRC13812) of *V. harveyi* [8]. More importantly, according to the protective evaluation in the present study, the protective immunity raised by one shot of PLG-PLE/rGAPDH MPs protected 80% of grouper from a lethal peritoneal challenge caused by the heterologous, highly virulent Vh MML-1 strain of *V. harveyi* and enabled grouper to survive at least 28 days following the experimental challenge (Figure 4). Under PLE adjuvant effect and PLG encapsulation, the vaccine antigen, rGAPDH, produced from the lowly virulent strain exerted effective cross-protection against the highly virulent strain. In further comparison, following single immunization, PLG-PLE/rGAPDH MPs provided a significantly higher survival rate (80%) in fish than the oil formulation containing inactivated bacteria (60%). In addition, we believe that the PLE-induced adjuvant effect, which has been shown in our previous study [8], should also contribute to the GAPDH-specific protective immunity against *V. harveyi* in grouper. The conspicuous vaccine efficacy found in the present study therefore has indicated that the sustained retention of both PLE peptide and rGAPDH protein offers an essential function to induce protective immunity against *V. harveyi*. On the other hand, a low relative percentage survival (40%) induced by GAPDH in large yellow croakers (*Pseudosciaena crocea*) after a lethal *V. harveyi* challenge has been previously shown by Zhang and his coauthors [9,10]. Despite the differences in fish species and pathogenic bacterial strains, in comparison, the PLG MP vaccine prepared in the present study can afford a much higher protection rate (80%) than GAPDH alone proposed by Zhang and his coauthors. Thus, PLG-PLE/rGAPDH MPs could be applied in developing an efficacious single-dose vaccine against *V. harveyi* for future farm use after enhancement of the load of PLE and rGAPDH in PLG MPs and optimization of stable release of PLE and rGAPDH [40].

## 5. Conclusions

We have successfully extended our previous finding to assess the feasibility of the development of a single-dose vaccine based on the PLG encapsulation technique that is promised to undertake the sustained release of both rGAPDH and PLE from PLG MPs. Following single peritoneal vaccination in grouper, the sustained release of both PLE peptide and rGAPDH protein from PLG-PLE/rGAPDH MPs results in not only long-lasting immunity (12 weeks) but also strengthened protection against *V. harveyi*. Therefore, PLG-PLE/rGAPDH MPs could be applied in developing an efficacious single-dose vaccine against *V. harveyi* for future farm use.

## Figures and Tables

**Table 1:** Primers used in real-time PCR.

Primer Name	Nucleotide Sequence (5′ → 3′)	Amplification Size (bp)	Accession Number/Reference
TNF-α (F)	GTGTCCTGCTGTTTGCTTGGTA	207	FJ009049/[29]
TNF-α (R)	CAGTGTCCGACTTGATTAGTGCTT		
β actin (F)	TGCTGTCCCTGTATGCCTCT	225	AY510710/[30]
β actin (R)	CCTTGATGTCACGCACGAT		

**Table 2:** TNF-α production of head kidney lymphocyte cultures from vaccinated grouper.

Group ^a^	TNF-α (Fold Change) ^b^	
	Lysate	Con A
PLG-PLE/rGAPDH MPs	12.4 ± 1.5 ^c^	2.7 ± 0.9 ^f^
Inactivated bacteria (ISA 763 AVG)	3.2 ± 0.5 ^d^	2.8 ± 0.6 ^f^
rGAPDH	1.4 ± 0.3 ^e^	2.7 ± 0.7 ^f^
PLE	1.2 ± 0.4 ^e^	2.6 ± 0.5 ^f^
PLG empty MPs	1.1 ± 0.2 ^e^	2.5 ± 0.7 ^f^
PBS	0.9 ± 0.3 ^e^	2.4 ± 0.5 ^f^

^a^ Twelve weeks after peritoneal vaccination, lymphocytes from different fish groups were stimulated with bacterial lysate (20 μg/mL) or Con A (10 μg/mL). Real-time PCR was undertaken to analyze TNF-α production as described in Section 2.9. ^b^ The fold change was counted by dividing the quantity of TNF-α mRNA from stimulated cells by the quantity of TNF-α mRNA from unstimulated cells. ^c,d,e,f^ A significant difference (*p* < 0.05) exists between groups with different superscript letters.
